# Ewha Medical Journal passed the scientific evaluation by PubMed Central and succeeded in being included in DOAJ, but failed to be accepted by Scopus

**DOI:** 10.12771/emj.2025.00024

**Published:** 2025-02-18

**Authors:** Sun Huh

**Affiliations:** Institute of Medical Education, Hallym University College of Medicine, Chuncheon, Korea

## Passing PubMed Central’s evaluation for scientific quality

**Fig. 1.**
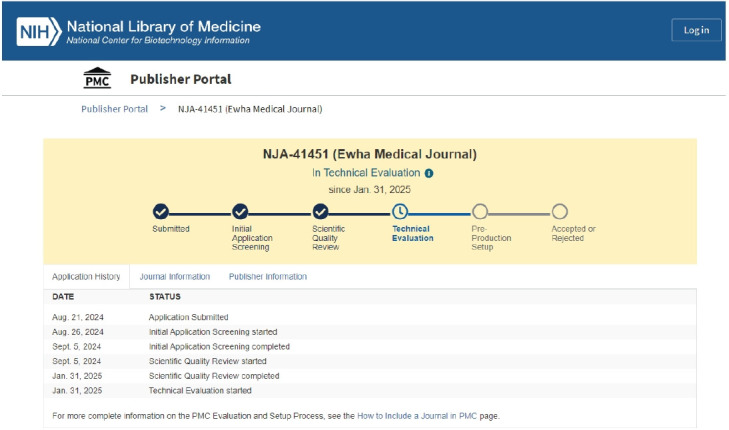
Evaluation phase status found in the PMC Publisher Portal [cited 2025 Feb 1].

I was thrilled to receive an email on the morning of February 1, 2025 from the PubMed Central (PMC) Applications Team informing me that *Ewha Medical Journal has passed the Scientific Quality Review by NLM for PMC, and PMC will take content back to 2023*. Because the technical evaluation phase remains ongoing, our management team is committed to meeting the technical evaluation criteria for full-text Journal Article Tag Suite (JATS) XML files [1]. This evaluation status is also visible on the PMC Publisher Portal (Fig. 1) [2].

Feedback from PMC reviewers on March 12, 2022, identified several issues, including article quality, study design, methods description, adherence to guidelines, clarity of writing, conflict of interest statements, and diversity among authors and editors [3]. We resubmitted our application to PMC on August 21, 2024. Before submission, the management team meticulously verified the scientific quality of each article. The following efforts were undertaken to address the PMC reviewers’ comments:

First, beginning with the October 2023 issue, the study design has been explicitly indicated in each article title.

Second, manuscripts are now structured according to the appropriate reporting guidelines for their respective study designs. For example, we employed the PRISMA statement for systematic reviews, the CONSORT statement for randomized controlled trials, the STROBE statement for observational studies, the COREQ statement for qualitative studies, and the CARE statement for case reports—all available at https://www.equator-network.org/.

Third, the methods sections of all original articles have been revised to align with the corresponding reporting guidelines. Each article now clearly details the study design, setting, participants, data sources and measurement, variables, potential biases, sample size, and statistical analysis.

Fourth, all manuscripts have been proofread by professional native English speakers to eliminate unfamiliar or ambiguous expressions.

Fifth, a conflict of interest statement has been included in every article.

Sixth, our editorial board now represents three continents—Asia (Korea), Oceania (Australia), and North America (United States). In addition, since 2022 we have attracted contributions from an internationally diverse group of authors, including those from China, Indonesia, Thailand, Turkey, Iran, Australia, the United States, Bulgaria, Denmark, Germany, the Netherlands, Switzerland, and the United Kingdom, even though our primary regional focus remains Korea.

We hope that the reviewers will recognize these efforts. We are pleased to have passed the scientific evaluation on our second application. This achievement was made possible with the full financial support and encouragement of Dr. Eun Hee Ha, Dean of the Ewha Womans University College of Medicine (August 2021–January 2025).Many medical journals in Korea have been included in PMC since 2008 [4]. As of February 1, 2025, 165 journals from Korea were found with the search term “journalspmc AND Korea [country]” in the NLM Catalogue at https://www.ncbi.nlm.nih.gov/nlmcatalog (Supplement 1). Of these, 21 journals appeared as duplicates due to title changes, leaving 144 unique journals included in PMC. Additionally, some journals are published by organizations located outside Korea, so the total number of Korean journals in PMC is even higher. Both Korean and English journals—such as the *Ewha Medical Journal, the Journal of the Korean Society of Radiology, Women’s Health Nursing, and Ŭi sahak (Korean Journal of Medical History*)—have been indexed in PMC [5]. Being a PMC journal and searchable in PubMed is expected to attract manuscript submissions from researchers worldwide, even though our journal’s primary focus remains on Korea [6].

## Being accepted in DOAJ

**Fig. 2.**
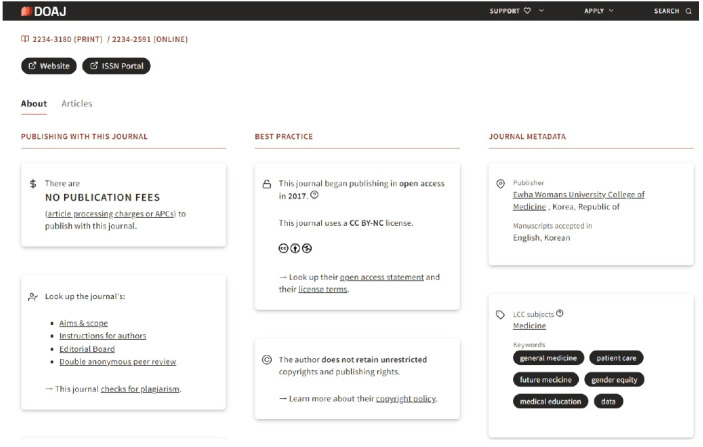
Dashboard of the *Ewha Medical Journal* found at https://doaj.org/toc/2234-2591.

Another piece of good news is that the *Ewha Medical Journal* has been included in the Directory of Open Access Journals (DOAJ). I received a message from DOAJ stating that *the application submitted for the Ewha Medical Journal on August 24, 2024, has been accepted for inclusion in DOAJ* on November 24, 2024. The journal’s dashboard is found at https://doaj.org/toc/2234-2591 (Fig. 2). Being listed in DOAJ signifies international recognition as a high-quality open-access journal that meets strict evaluation criteria [7].

## Failure to be listed in Scopus

Unfortunately, on January 6, 2025, we received disappointing news from the Scopus Title Evaluation Team—namely, *the Content Selection and Advisory Board (CSAB) has advised not to accept the title for Scopus inclusion at present* (Supplement 2). The main reasons cited were the low annual publication volume, the presence of multiple subject areas without clear cohesion, contributions from outside Korea, and a perceived lack of focus in the editorial strategy. We may reapply on January 6, 2027, or later. Although these comments are disheartening, our editorial team remains committed to enhancing the journal in response to the CSAB’s feedback.

## Role of the editor-publisher of society journals

Typically, journal editors at commercial publishing companies focus solely on peer review, while all other editing and publishing tasks are handled by the publisher. These tasks are supported by professional management teams—including managing editors, manuscript editors, copy editors, ethics editors, legal consultants, statistical editors, language editors, layout editors, graphic designers, and information technology engineers who produce full-text XML files, manage websites, and maintain submission systems. In contrast, the editor-publisher of a society journal must be familiar with the entire editing and publishing process. Overseeing applications to international literature databases is also a crucial responsibility for the editor-publisher. Fully understanding and meeting all evaluation criteria for editing and publishing is challenging, and there are no shortcuts. Our editors and management team at the Ewha Medical Journal are committed to upholding international standards of scientific rigor in both style and format.

## Change of publishing company

Starting in February 2025, M2PI (https://www.m2-pi.com/), a top-tier publishing company that has produced 210 scientific, technological, engineering, and medical journals in Korea, will take over publishing for the *Ewha Medical Journal*. Since 2020, GuhMok (https://www.guhmok.com/) has provided excellent editing and publishing support. I appreciate the sincere and high-quality work of Mr. Yeon-Wook Kim and his team. The change in publishing company is intended to introduce more sophisticated journal metrics, enhanced search technologies, and unique presentation features.

## Provision of templates for reporting guidelines of common study design

Starting in February 2024, we will provide templates for reporting guidelines to assist authors. Using these templates will simplify manuscript preparation and enable reviewers to quickly assess the scientific quality of submissions. Templates will be available for systematic reviews/meta-analyses, randomized controlled trials, non-randomized controlled trials, before-and-after studies, observational studies, diagnostic studies, qualitative studies, and case reports. For other study designs, authors may refer to the Equator Network (https://www.equator-network.org/) and consult with an editor before submission. Most clinical articles from Korea are observational studies—such as cohort, case-control, or cross-sectional studies—and the Korean translation of the STROBE statement was provided in the October 2024 issue of the journal [8].

I am pleased to share the successful applications to three databases [3]. However, the challenge of gaining inclusion in Scopus remains. I hope that the change in our publishing partner and the implementation of reporting guideline templates will serve as a turning point in elevating the journal to a top-tier level.

## Supplementary materials

Supplementary files are available from Harvard Dataverse: https://doi.org/10.7910/DVN/JSBQ32

Supplement 1. 165 journals in NLM Catalog (https://www.ncbi.nlm.nih.gov/nlmcatalog) found with the search term, “journalspmc AND Korea [country]”.

Supplement 2. Letter from Scopus Title Evaluation team.
