# Supplementary material for: Ewha Medical Journal passed the scientific evaluation by PubMed Central and succeeded in being included in DOAJ, but failed to be accepted by Scopus
Source: Ewha Med J. 2025 Feb 18;48(2):e21. doi: 10.12771/emj.2025.00024 (PMC12277496; doi:10.12771/emj.2025.00024)
Supplement: Supplementary file 2 — Supplement 2. Letter from Scopus Title Evaluation team. [file emj-2025-00024-suppl2.pdf]

Supplement 2. Letter from Scopus Title Evaluation team

The review of your title for Scopus is complete

Sendr: Scopus Title Evaluation Team :2025.01.06 (Mon) 23:01
Receiver: Sun Huh

Title: Ewha Medical Journal
ISSN / E-ISSN: 2234-3180 / 2234-2591
Publisher: Ewha Womans University College of Medicine

Dear Dr. Sun Huh,

The title mentioned above has been evaluated for inclusion in Scopus by the Content Selection & Advisory Board (CSAB).

The review of this title is now complete and the CSAB has advised to not accept the title for Scopus inclusion at the present time. For your information, the reviewer comments are copied below:

This mono-institutional journal from the Ewha Womans University College of Medicine in South Korea has been in print since 1978.

The stated aims unusually include intent "to contribute to improving and serving human society based on the Christian values of education, truth, goodness, and beauty", suggesting that it has a faith based element.

It has been in print since 1978, over which time it has secured some 250 citations. However, it is only publishing fewer than 30 items per annum from many different subject areas, with no cohesion or structure to the content.

Moreover, a number of articles are not sourced from South Korea, and the overall impression is given of a serious lack of coherence or focus in the editorial strategy.

Therefore, regrettably, I take the view that this journal would represent a reputational risk to SCOPUS in its present form,.

It is displaying no safeguards to the uncontrolled influx of articles which a SCOPUS listing often attracts.

This journal would benefit from a clear strategic focus on Editorial policy and South Korean content which could be judged to be consistent with its local institutional origins.

The widely varying subject content should be consistently presented in distinct subject areas around understandable themes.

If in the future these comments are addressed, you may decide to submit a new application at any time after the following date: January 2027.

At that time, you will be required to upload a cover letter detailing how the above comments have been addressed.

A full review will be conducted upon resubmission and having addressed all the abovementioned concerns will therefore not guarantee acceptance.

Finally, we strongly advise you to read through our FAQ:

Helping to improve the Scopus submission & success process for editors and publishers.

Practical information for journal editors can be found in our Role of an editor FAQ.

Yours sincerely,

Scopus Title Evaluation Team
titlesuggestion@scopus.com

Notice: Scopus is owned by Elsevier B.V. and Elsevier is solely responsible for the content selection policy of Scopus. In order to come to a decision to accept or reject a title for Scopus, Elsevier follows the independent advice from the Scopus Content Selection & Advisory Board (CSAB). However, Elsevier in consultation with the CSAB reserves the right to change decisions, adjust the selection criteria, or re-evaluate titles that are accepted for Scopus without prior notice. Decisions made by the CSAB do not guarantee inclusion into Scopus due to not being able to come to terms with the publisher on a licensing agreement. In no event shall Elsevier be liable for any indirect, incidental, special, consequential or punitive damages arising out of or in connection with any advice disclosed or any selection decision made. This statement must not be used for advertisement purposes.
